# Investigation on the Effect of Calcium on the Properties of Geopolymer Prepared from Uncalcined Coal Gangue

**DOI:** 10.3390/polym15051241

**Published:** 2023-02-28

**Authors:** Qingping Wang, Longtao Zhu, Chunyang Lu, Yuxin Liu, Qingbo Yu, Shuai Chen

**Affiliations:** 1 School of Materials Science and Engineering, Anhui University of Science and Technology, Huainan 232001, China; 2 State Key Laboratory of Mining Response and Disaster Prevention and Control in Deep Coal Mines, Anhui University of Science and Technology, Huainan 232001, China; 3 Anhui Generic Technology Research Center for New Materials from Coal-Based Solid Wastes, Anhui University of Science and Technology, Huainan 232001, China

**Keywords:** calcium hydroxide, coal gangue, geopolymer, response surface method, alkali activator

## Abstract

In this paper, the influence of calcium on coal gangue and fly ash geopolymer is explored, and the problem of low utilization of unburned coal gangue is analyzed and solved. The experiment took uncalcined coal gangue and fly ash as raw materials, and a regression model was developed with the response surface methodology. The independent variables were the CG content, alkali activator concentration, and Ca(OH)_2_ to NaOH ratio (CH/SH). The response target value was the coal gangue and fly-ash geopolymer compressive strength. The compressive strength tests and the regression model obtained by the response surface methodology showed that the coal gangue and fly ash geopolymer prepared with the content of uncalcined coal gangue is 30%, alkali activator content of 15%, and the value of CH/SH is 1.727 had a dense structure and better performance. The microscopic results demonstrated that the uncalcined coal gangue structure is destroyed under an alkali activator’s action, and a dense microstructure is formed based on C(N)-A-S-H and C-S-H gel, which provides a reasonable basis for the preparation of geopolymers from the uncalcined coal gangue.

## 1. Introduction

China is one of the countries with the largest coal reserves and produces the highest amount of coal annually. Coal will be the country’s primary energy pillar in the future, but its mining process yields large quantities of solid waste. China currently has over 7 billion tons of coal gangue (CG) and more than 2600 large-scale CG hills [1]. Due to these severe pollution problems, many researchers are searching for materials that can effectively utilize CG; geopolymers have been proposed as a suitable inorganic binder because of their excellent durability, mechanical strength [2], and large consumption of CG [3,4]. Alumina and silica are the main elemental components of CG, and the main mineral components are quartz, kaolinite, and muscovite. Compared with the high pozzolanic property of fly ash [5], CG without special treatment has a stable structure and low activity. In the use of CG, its pozzolanic properties should be maximized through activator. The internal crystal phase composition of CG can be transformed via calcination at 700–900 °C [6,7]. Experimental findings obtained under different reaction conditions [8] have shown that an appropriate calcination temperature and time are needed to destroy the structures of kaolinite and quartz and improve the hydration reactivity of CG [9,10]. Raw CG is calcined at 550 °C to convert the kaolinite in its structure into metakaolin, which is then converted into mullite at 950 °C [11].

According to its calcium content, CG can be classified as high-, medium-, or low-calcium gangue. Compared with alkali-activated slag, geopolymers prepared by calcining CG have poor strength and other properties, with their differences in calcium content being the main factor affecting the performance discrepancy between the two materials [12]. Lime or red mud and other materials can be added during the calcination of CG to increase its calcium content and thus enhance its activation efficiency. Li et al. [6] effectively improved the compressive strength of geopolymers by adding CaSO_4_ and CaO as activation additives during CG calcination. Mineralizers, such as fluorite and gypsum, can also promote CG activation during calcination [13]. Compared with the commonly used high-calcium mineral admixtures, geopolymers exhibit denser structures, higher bulk densities, and better compressive strength [14,15]. Chen et al. [16] used CaO and SO_3_ as admixtures to promote the geopolymer reaction, thereby increasing the number of gel products and compensating for the shrinkage of the geopolymer.

Due to considerable research data and activation processes, high-calcium additives can be added during the preparation of CG-based geopolymers to obtain ideal properties. However, researchers should focus on the energy consumption and environmental impact of geopolymers, a new gel material, before using them to replace traditional cement and other gel materials. CG calcination consumes large amounts of energy, so uncalcined CG (UCG) should be used to prepare geopolymers. Through experiments, Geng et al. [17] found that UCG can be mixed with red mud to prepare geopolymers with excellent development strength. Guo et al. [18] prepared suitable geopolymer grouting materials by compounding UCG, fly ash (FA), and slag. Preparing geopolymers from UCG is significant for energy consumption reduction and the environment.

To sum up, high-strength geopolymers can be prepared by mixing calcined coal gangue with fly ash and other raw materials. However, the energy consumption in the process of coal gangue calcination is an important factor limiting the utilization of coal gangue. In this study, UCG was used as the mineral raw material and mixed with FA to prepare geopolymer (CG–FA geopolymer (CFG)); the effect of calcium on the mechanical properties and microstructure of geopolymer was investigated by using the mixture of calcium hydroxide and sodium hydroxide as an activator. Desulfurization gypsum and a water-reducing agent were used as admixtures to adjust the compressive strength of the geopolymer. The response surface methodology (RSM) was chosen as the experimental design to optimize the factors that influence the optimization of geopolymer properties, and a multifactor, multiresponse collaborative optimization method was adopted. The CG content, alkali activator content, and Ca(OH)_2_ to NaOH ratio (CH/SH) were the independent variables, and the compressive strengths for different curing periods were the target values. Relevant models were established to analyze the other, different experimental conditions. The CFG microstructure was examined via XRD and SEM–EDS. FTIR and ^29^Si nuclear magnetic resonance (NMR) was used to characterize the changes in the chemical bonds and degrees of polymerization within the CFG structure.

## 2. Experimental

### 2.1. Materials Selection and Pretreatment

**Figure 1:**
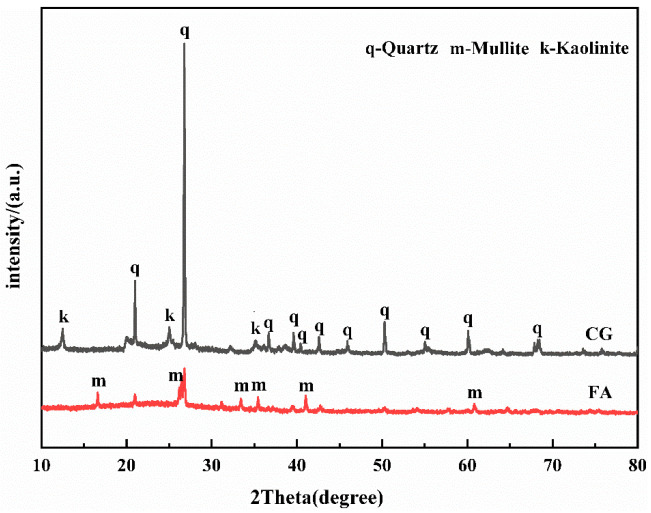
The XRD patterns of raw material.

**Figure 2:**
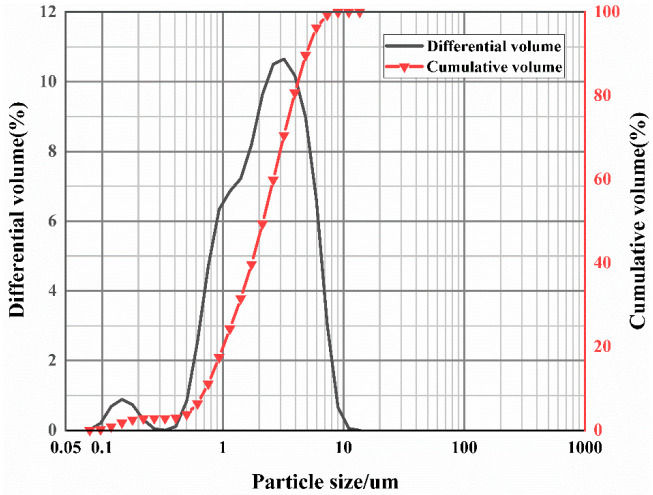
Particle size distributions of coal gangue.

The chemical composition of UCG and FA is shown in Table 1. It can be seen from the table that UCG and FA contain a large amount of SiO_2_ and Al_2_O_3_. Therefore, UCG and FA were selected as the silicon and aluminum raw materials. The coal gangue used in the experiment was obtained from Xingtai (Hebei, China). The X-ray diffraction (XRD) analysis and microstructure analysis is shown in Figure 1 and Figure 2, which demonstrates that there are a large number of mineral structures such as kaolinite and quartz with stable structures in the original coal gangue. The fly ash was obtained from the Henan Datang Power Plant. Table 1 shows that the UCG and FA contain a large amount of SiO_2_ and Al_2_O_3_, but the calcium oxide content of coal gangue is less than 3%, which belongs to low calcium coal gangue. Additive selection desulfurization gypsum (purity ≥ 93%). The alkali activator is prepared by blending sodium hydroxide and calcium hydroxide (analytical grade, purity ≥ 98%). The activator must be mixed with water and cooled to room temperature before use.

The massive coal gangue shall be pretreated before use. First, use a hammer to crush the large pieces of raw coal gangue, and then put the lumpy coal gangue into the roller ball mill to crush it into coarse aggregate. After 120 min, grind the material with a planetary mill and sieve it to prepare fine coal gangue particles. The particle size distribution of coal gangue after ball milling is shown in Figure 2. The ground coal gangue can increase the specific surface area of the particles, significantly improve the gel activity, and facilitate the dissolution of the reaction process, which is the basis for the preparation of geopolymers.

### 2.2. Specimen Preparation and Experimental Design

In this study, based on the response surface design experiment, three main factors were selected to control the performance of geopolymer, and a total of 15 experiments were carried out. The different levels of the three independent variables are shown in Table 2. The content of coal gangue was selected as factor A, the activator which has an essential influence on the properties of silicon-alumina raw materials was chosen as factor B, and Ca(OH)_2_ to NaOH ratio (CH/SH) was selected as factor C. The solid–liquid ratio was fixed at 0.7, and the additional amount of desulfurization gypsum was 12% of the solid waste silica-alumina material.

According to the experimental conditions, an appropriate amount of sodium hydroxide was dissolved in water with calcium hydroxide powder and allowed to stand for 12 h. The activator and raw materials were mixed in a pure slurry mixer at 1000~1200 r/min for 15 min. The mixed slurry was poured into a 40 mm × 40 mm × 40 mm six-joint mold. Then, the mold wrapped with plastic film was placed into a high temperature curing box at 80 °C for 24 h. The sample taken from the mold was placed in a curing box at 25 ± 1 °C and 95% relative humidity.

### 2.3. Macroscopic Test and Microstructure Characterization

The CFG macroscopic properties were tested in compressive strength: the experimental data were tested according to GB/T 17671-2020 by an automatic cement constant-force test machine.

The chemical bonds of the geopolymer were characterized in the reaction process by Fourier Transform Infrared Spectroscopy (FT-IR) (Nicolet380). The type and composition of the polymer product was determined by SEM (FlexSEM1000, Hitachi, Hong Kong, China) by observing the microstructure and morphology of the material. An X-ray diffractometer examined the mineral composition. The sample scanning speed was controlled at 5°/min, and the scanning range was 5~80° (2θ°). The chemical shifts of ^29^Si NMR samples were tested using a spectrometer. The Gaussian linear peak was fitted by PeakFit v4.0 software to obtain the relevant result.

## 3. Results and Discussion

### 3.1. Validation and Analysis of ANOVA Model

Table 3 lists the experimental compressive strengths of the geopolymer samples for different curing periods and analysis results obtained from the Design-Expert software. An analysis of the nonlinear fit of models shows that the second-order model is the most effective. The specific regression model for compressive strength (Y) is shown in Equations (1) and (2).

(1) $$Y_{7d}=5.47\;-\;1.16A+0.77B+0.96C\;-\;0.72\mathrm{AB}\;-\;0.15\mathrm{AC}\;-\;3.23\mathrm{BC}+1{.82A}^{2}+1{.84B}^{2}\;-\;0{.13C}^{2}$$

(2) $$Y_{28d}=11.87\;-\;3.44A\;-\;0.94B+1.3C+2.7\mathrm{AB}+0.42\mathrm{AC}\;-\;3.18\mathrm{BC}+2{.09A}^{2}+0{.19B}^{2}\;-\;3{.18C}^{2}$$

where A is the CG content, B is the alkali activator content, and C is the CH/SH value.

The relationship between the 7- and 28-day-curing compressive strength results and the independent variables were analyzed using RSM. Their coefficients of variation were 7.53% and 9.05% (<10%) [19], and the feasibility of the equation analysis was verified. The *p* value can be used to express the effectiveness of the hypothesis and mismatch test analysis during the analysis; the *p* value between 0.05 and 0.1 is significant, and that below 0.05 is very substantial [20]. Table 4 lists the results of the ANOVA analysis of the regression model. The *p* values of the factors are less than 0.05, showing that the regression effect is significant [21], whereas the *p* values of the interaction terms are all less than 0.05, indicating that the partial *p* values are significant. The test reliability of the polynomial equations is tested using R^2^ values. As seen in Table 5, the R^2^ values of the 7- and 28-day-curing compressive strength models are 0.9834 and 0.9787, respectively.

### 3.2. Influence of Various Factors on Compressive Strength

**Figure 3:**
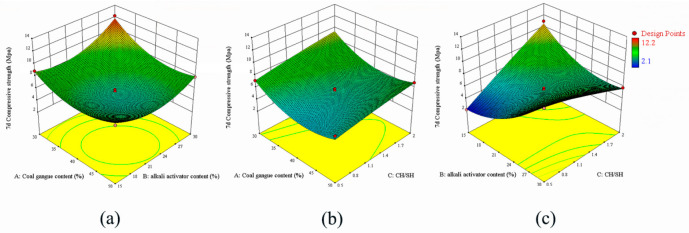
Response surface model of 7 day curing compressive strength, (**a**) CG content vs. alkali activator content, (**b**) CG content vs. CH/SH, (**c**) alkali activator content vs. CH/SH.

The influence of A, B, and C on the 7-day-curing compressive strength is shown in Figure 3. Figure 3a reveals an interaction between A and B. When the value of A is 30, a large amount of FA enhances the reactivity of the raw material. As the B value increases, the concentration of the alkali activator increases, thereby accelerating the dissolution of the raw material structure. The relationship between A and C in Figure 3b shows that as the value of C increases, the dissolution of the raw material structure accelerates, and the calcium ions in the reaction process react with the silicon–oxygen tetrahedra to form a gel structure, which improves the 7d compressive strength. As shown in Figure 3c, with a decrease in the B content, the slurry strength decreases and then increases. As C gradually decreases, the compressive strength increases significantly. However, when C is 2, the 7-day-curing compressive strength decreases slightly with the B value. This is because when C is high, with the increase in the B value, more calcium ions react with the silica tetrahedron to improve the compressive strength and carbonization occurs at the same time. By contrast, excess calcium ions will carbonize with carbon dioxide in the air to form carbides, such as calcite, the reduction in calcium content will reduce the reaction of active silicon–alumina materials and decrease the 28d strength growth.

**Figure 4:**
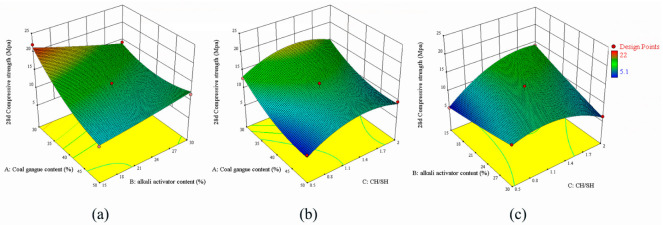
Response surface model of 28 day curing compressive strength, (**a**) CG content vs. alkali activator content, (**b**) CG content vs. CH/SH, (**c**) alkali activator content vs. CH/SH.

Figure 4 shows the response surface of the effects of A, B, and C on the 28-day-curing compressive strengths of the specimens. Figure 4a shows that the experimental results of the slurry decrease with an increase in A because the large quantity of impurities and more structurally stable quartz and kaolinite structures in the UCG reduce the structural compactness of the geopolymer. The correspondence between A and B in Figure 4a suggests that the slurry strength is high when B is about 10%. With a gradual increase in alkali activator concentration, more unreacted sulfate radicals and alkaline cations will remain in the structure in the later stages of the reaction, which will corrode the material structure and reduce the structural strength. As shown in Figure 4c, compressive strength increases with C when the latter is below 1.1. However, when the C value is higher than 1.1, the compressive strength decreases. This is because with an increase in calcium concentration, the gel phase increases and the polymerization degree of the material increases. Still, an excessively high calcium concentration will accelerate the reaction early, thus reducing the calcium content in the later reaction stages and decreasing the gelation in the subsequent curing process. Part of the calcite structure generated in the early stage will also dissolve in the later reaction stages, thereby reducing the gel structure and strength.

After the influence of each independent variable on compressive strength was evaluated, the maximum 28-day curing compressive strengths of the geopolymer were regarded as the optimal values. The optimized conditions are as follows: 30% CG content, 15% alkali activator content, 1.727 CH/SH value, and the results of compressive strength predictions for 7-day curing (11.494 MPa) and 28-day curing (22.513 MPa). Experiments were performed using these optimal ratios, and the experimental 7- and 28-day curing compressive strengths are 11 MPa and 20.5 MPa, respectively. The error between the specimens’ compressive strengths in the experiments and the model’s predicted values is less than 10%, which means that the numerical model has high accuracy.

### 3.3. FTIR Spectroscopic and Mineral Morphology Analysis

**Figure 5:**
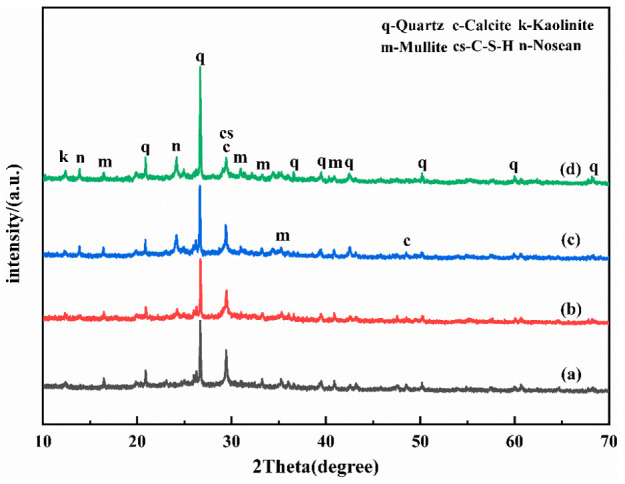
The XRD patterns of CFG at 28d, (**a**) Group1, (**b**) Group3, (**c**) Group11, (**d**) Group6.

**Figure 6:**
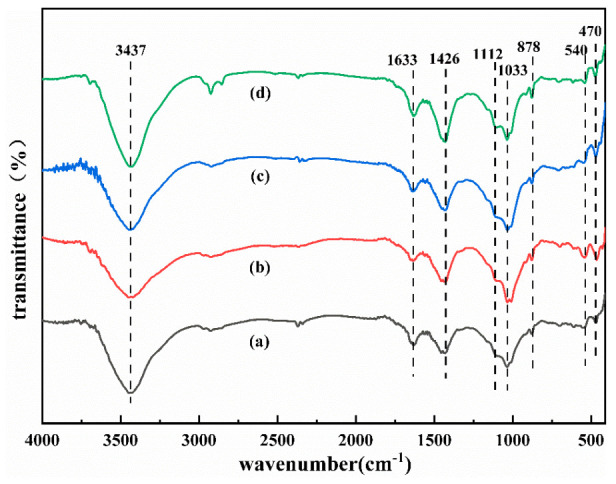
IR analysis of the CFG at 28d, (**a**) Group 1, (**b**) Group 3, (**c**) Group 11, (**d**) Group 6.

Figure 5 shows the XRD results for the geopolymer paste samples in Table 3. According to Figure 6, the absorption peak of kaolinite in CG almost disappears. The diffraction peak of quartz still exists but has a reduced intensity, indicating that the structure of the crystal phase of CG has been destroyed under the action of the composite alkali activator. A broad hump is observed in the 2θ range of 22–35°, indicating the presence of C–S–H and C–(N)A–S–H gels [22]. As the A value increases, the diffraction peak intensities of quartz and kaolinite also increase, mainly because the activation efficiency of the UCG decreases [23]. The unreacted coal gangue particles can build up and destroy the integrity of the structure, resulting in reduced strength. As the B value increases, the increase in calcium content promotes the formation of C–S–H in the early stage and improves the early strength. The 2θ characteristic peaks at 14° and 29° indicate the presence of a nosean. The excessive c value increases the sodium content in the reaction precursor, and the excessive sodium reacts with gypsum to form nosean which accumulates in the material structure, damaging the performance of the geopolymer.

Figure 6 depicts the FTIR spectra of different samples on day 28. The bands at 3440 cm^−1^ and 1650 cm^−1^ can be attributed to the tensile and bending vibrations of H–O–H in the molecular water [24]. The stretching vibration peak at 1450 cm^−1^ can be attributed to the tensile vibrations of the sample’s O–C–O bonds. The characteristic infrared absorption peaks of geopolymers are usually distributed within 900–1300 cm^−1^, which is related to Si–O–T (T = Si, Al) asymmetric stretching [25]. The stretching vibration peak from 1009 to 1030 cm^−1^ is related to the asymmetric stretching of the Si–O–Si (Al) bond of the C–(N)A–S–H gel. The vibrational peak at 790 cm^−1^ is associated with quartz [26]. The bands around 460 cm^−1^ are associated with the symmetrical stretching vibrations of Si–O–Si, which may be associated with kaolinite [27]. As the A value decreases, the corresponding absorption peak wave numbers at 460 cm^−1^ and 539 cm^−1^ gradually decrease while moving higher. The absorption peak at 1450 cm^−1^ decreases with increases in the B and C values, indicating that CG does not fully participate in the reaction, which is consistent with the XRD analysis results.

### 3.4. Microstructure Analysis

**Figure 7:**
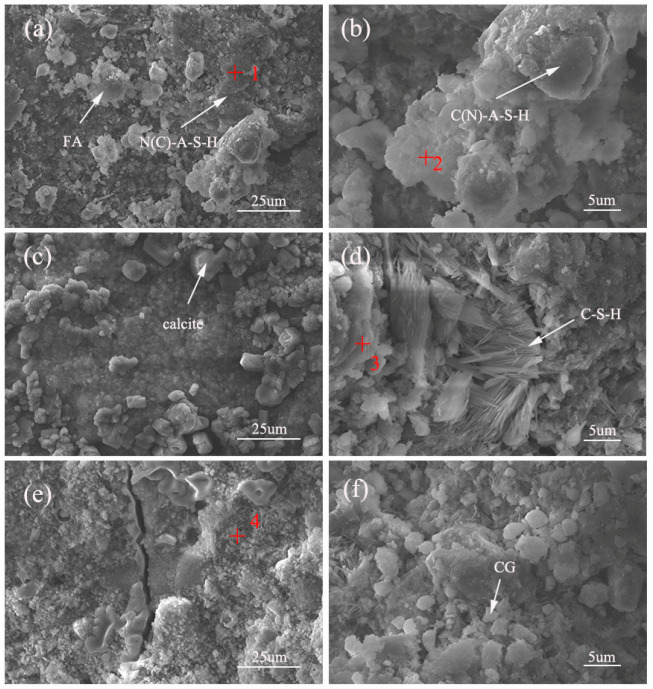
The SEM images of the CFG at 28d of Group 1 (**a**,**b**), Group 11 (**c**,**d**) and Group 6 (**e**,**f**).

The surface morphology of the CFG was observed and analyzed using SEM–EDS. The microstructures of the samples and their EDS spectra under different experimental conditions are shown in Figure 7 and Table 6, respectively. As shown in Figure 7a,d, large numbers of flocculent- and gel-like hydration products are generated in the reaction; the main components are C–N–A–S–H and C–S–H [28,29]. As shown in Figure 7c,d, there is also some calcite and C-S-H in the structure. Microstructure analysis of different samples shows that with a decrease in A, a new C(N)–A–S–H gel forms. This is because of an increase in the active silica–alumina substances participating in the reaction process, which increases the amount of gel product and enhances microstructure density. Moreover, the shrinkage in the geopolymer itself or the mechanical property test may have created microcracks in the sample [30]. Increases in the values of B and C lead to the accumulation of excess alkali cations and residual sulfate in the structure during the reaction, thus compromising the structure’s integrity, makes the microstructure loose, increases the pore structure, and reduces the mechanical strength.

### 3.5. ^29^Si NMR Analysis

**Figure 8:**
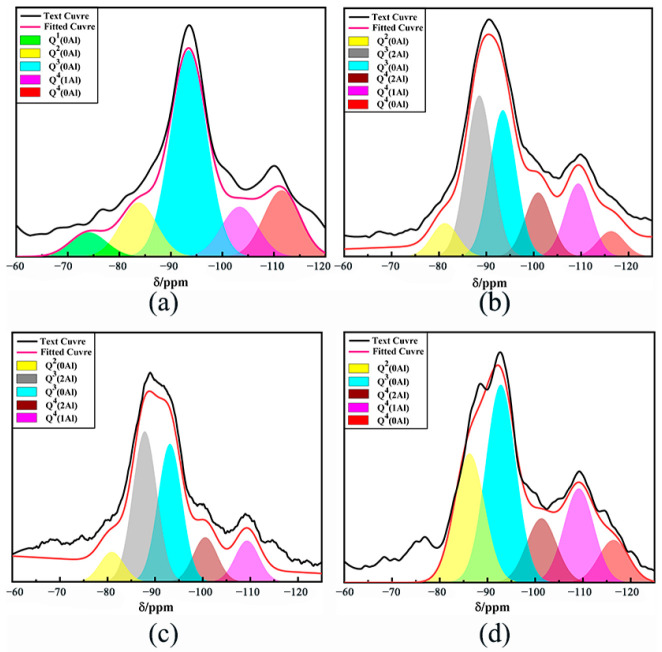
^29^Si NMR spectra of CFG at28d, (**a**) CG, (**b**) Group 1, (**c**) Group 11 and (**d**) Group 6.

In the structure of silicate mineral materials, each Si atom is generally surrounded by four O atoms to form a [SiO_4_]^4−^ tetrahedron, the basic structural unit of silicate [31]. In the ^29^Si NMR test analysis, the different ^29^Si NMR signals corresponding to these five tetrahedral backbones represent the silicon–oxygen tetrahedron’s aggregation degree [32]. As seen in Figure 8 and Table 7, the absorption peak in the CG raw material is mainly from the Q_0_^3^ structural unit from kaolin (at −93.59 ppm) and the Q_0_^4^ structural unit from quartz (at −110.14 ppm) [33]. Compared with the findings in Figure 8a,b, Q_0_^3^ and Q_0_^4^ move to a lower chemical shift when the strength decreases during the reaction between the alkali activator and CG. Under the action of the mixed alkali activator, the quartz and kaolin in CG decrease, and the polymerization degree in the sample increases. As depicted in Figure 8b–d, the peak strengths of quartz and kaolinite at −190 ppm and −109 ppm increase with A. When A increases to 50, the chemical shift at −92 ppm reappears, consistent with the XRD analysis results. The large intensity peak from −83 to −87 ppm comes from the Q^4^ structural unit of N–A–S–H or N (C)–A–S–H [34]. As shown in Table 7, with increases in B and C, the relative areas of Q_0_^3^ and Q_0_^4^ decrease and then increase. The relative area of Q_2_^4^ decreases gradually, indicating that the gradual decline in the network polymerization degree of reaction products leads to a reduction in compressive strength.

### 3.6. Analysis of Geopolymerization Process

**Figure 9:**
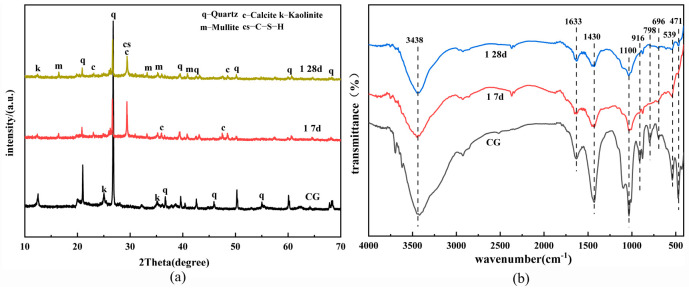
XRD (**a**) and IR (**b**) analysis of the CFG.

**Figure 10:**
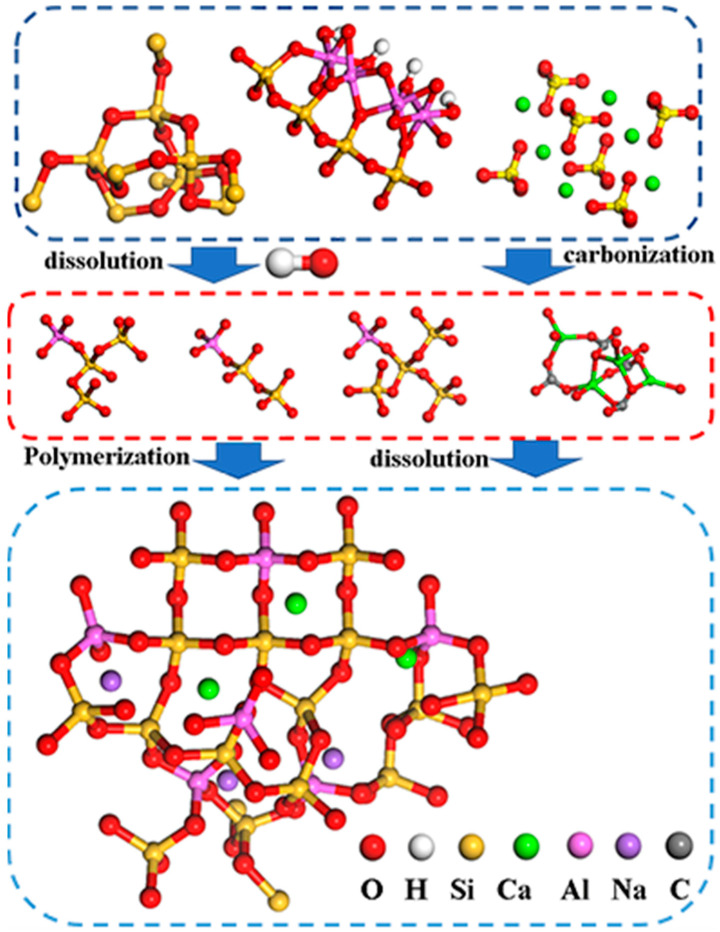
Mechanism diagram of possible bond formation in the reaction between UCG and gypsum.

Figure 9 shows the XRD spectra and infrared vibration bands of sample 1 on the 7th and 28th days and the unreacted CG, respectively. The peak of 2θ at 29° corresponds to calcite and C-S-H structure. The calcium content in the structure at the early stage of the reaction is high, which promotes the calcite formation at the early stage and improves the 7d compressive strength [35]. However, as curing progresses, the peak strength of CaCO_3_ structure decreases [36]. Figure 6 shows that the stretching vibration peak at 1450 cm^−1^ can be attributed to the tensile vibrations of the sample’s O-C-O bonds. The absorption peak broadens and shifts higher as the reaction proceeds, indicating that the calcite structure has been decomposed, which is consistent with the XRD analysis findings (calcite (CaCO_3_)) [16]. The crystal structure and functional group analysis show that the quartz and kaolinite structures were decomposed in an alkaline environment at the early stage of the reaction. At the same time, the high concentration of calcium content was carbonized to generate calcite and C-S-H, which were partially decomposed in the subsequent reaction process. The mechanism diagram of possible bond formation of alkali-activated UCG is shown in Figure 10.

## 4. Conclusions

We created a geopolymer called CFG, which is environmentally friendly and has good economic value. UCG was used as the raw material, and desulfurization gypsum and an alkaline activator (essential for CG utilization and recovery) were used as ligands.

The composite alkali activator, prepared by mixing sodium hydroxide and calcium hydroxide while increasing the calcium content, was used to prepare the geopolymer from UCG. It has been found that the addition of unburned coal gangue was an important factor affecting the compressive strength of geopolymer by optimizing the ratio. With the increase in coal gangue content, the compressive strength decreased. When the content of coal gangue was 30%, the maximum 28-day compressive strength was 22 MPa. The microstructure characterization and analysis showed that the inert structure of UCG could be dissolved to a large extent under the action of a mixed activator of sodium hydroxide and calcium hydroxide. The dissolved coal gangue and fly ash formed new structures under the action of activators, such as C-S-H and calcite. Adding the proper Ca(OH)_2_ and desulfurization gypsum to provide additional calcium content improved the early compressive strength. It could also produce a composite gel structure of N (C)–A–S–H with high strength characteristics.

In this study, coal gangue and fly ash were used as raw materials to prepare geopolymer, and the effect of calcium on the formation of geopolymer was also discussed. The experiment provides an experimental basis for the development of UCG and has good economic value. Future research will continue to simplify the activation process of coal gangue in order to improve the utilization rate of coal gangue and reduce the cost of preparing geopolymer.

## Figures and Tables

**Table 1:** Chemical composition of raw material (%).

Materials	SiO_2_	Al_2_O_3_	Fe_2_O_3_	CaO	MgO	K_2_O	TiO_2_	Na_2_O	MnO_2_	P_2_O_5_
FA	59.61	28.85	3.82	3.03	1.02	1.77	1.77	0.78	0.06	0.13
CG	61.72	25.74	4.13	1.18	0.80	2.36	0.93	0.40	0.06	0.08

**Table 2:** Factors and levels in Box–Behnken design.

Independent Variable Factor	Coding and Level		
	−1	0	1
A, Coal gangue content	30%	40%	50%
B, Alkali activator content	15%	22.5%	30%
C, CH/SH	0.5	1.25	2

**Table 3:** Compressive strength test results of geopolymer.

Run	A (%)	B (%)	C	7d Compressive Strength (MPa)	28d Compressive Strength (MPa)
1	30	15	1.25	8.9	22
2	50	15	1.25	7.5	8.6
3	30	30	1.25	12.2	14.3
4	50	30	1.25	7.9	11.7
5	30	22.5	0.5	7.2	12.8
6	50	22.5	0.5	5.7	6.2
7	30	22.5	2	8.9	14.5
8	50	22.5	2	6.8	9.6
9	40	15	0.5	2.1	5.1
10	40	30	0.5	9.8	10
11	40	15	2	11	14.1
12	40	30	2	5.8	6.3
13	40	22.5	1.25	5.1	13.1
14	40	22.5	1.25	5.8	11
15	40	22.5	1.25	5.5	11.5

**Table 4:** Response surface test results of geopolymer.

Response	7 d Compressive Strength		28 d Compressive Strength	
	F-Value	*p*-Value	F-Value	*p*-Value
Model	32.82	0.0006	25.48	0.0012
A	35.35	0.0019	89.11	0.0002
B	15.71	0.0107	6.63	0.0498
C	24.23	0.0044	12.74	0.0160
AB	6.87	0.0470	27.49	0.0033
AC	0.29	0.6108	0.68	0.4468
BC	136.03	<0.0001	38.01	0.0016
A^2^	39.84	0.0015	15.23	0.0114
B^2^	40.95	0.0014	0.13	0.7353
C^2^	0.21	0.6626	35.27	0.0019
Lack of Fit		0.2319		0.5963

**Table 5:** Model reliability test analysis.

Group	Std. Dev./Mpa	R^2^	Adj R^2^	Pred R^2^	C.V./%	Adeq Precisior
Model Y_7d_	0.55	0.9834	0.9534	0.7706	7.53	21.205
Model Y_28d_	1.03	0.9787	0.9402	0.7917	9.05	18.892

**Table 6:** The atomic percentage of elemental composition at each spot in Figure 7.

Position	O%	Na%	Al%	Si%	S%	Ca%	Si/Al	Description
1	38.494	12.373	16.772	23.939	0	8.422	1.421	N(C)–A–S–H
2	41.284	4.74	10.792	27.743	1.245	14.196	2.7	C(N)–A–S–H
3	40.542	2.526	18.041	18.529	0	15.136	1	C–A–S–H
4	41.456	26.145	2.318	7.253	18.521	4.306	3.14	N–A–S–H

**Table 7:** Relative peak area of the fitted curve of ^29^Si NMR spectra (%).

Sample	Q_0_^1^	Q_0_^2^	Q_0_^3^	Q_2_^3^	Q_2_^4^	Q_1_^4^	Q_0_^4^
CG	6	13	45	0	0	27	8
1	0	7	29	32	13	14	5
11	0	8	34	37	11	10	0
6	0	24	38	0	12	18	8

## Data Availability

Data obtained as described.
